# Mapping the cattle industry in Brazil’s most dynamic cattle-ranching state: Slaughterhouses in Mato Grosso, 1967-2016

**DOI:** 10.1371/journal.pone.0215286

**Published:** 2019-04-30

**Authors:** Petterson Vale, Holly Gibbs, Ricardo Vale, Jacob Munger, Amintas Brandão, Matthew Christie, Eduardo Florence

**Affiliations:** 1 Nelson Institute for Environmental Studies, Center for Sustainability and the Global Environment (SAGE), University of Wisconsin-Madison, Madison (WI), United States of America; 2 Department of Economics, University of São Paulo, Ribeirão Preto (SP), Brazil; 3 Laboratory of Soils, State University of Mato Grosso, Alta Floresta (MT), Brazil; College of Agricultural Sciences, UNITED STATES

## Abstract

The state of Mato Grosso is Brazil’s agribusiness powerhouse with a cattle herd of 30.2 million head in 2017. With land use patterns heavily influenced by beef production, which requires substantial land inputs, the state is a key target for environmental conservation. Yet the spatial and temporal dynamics of slaughterhouses in Mato Grosso remain largely unknown due to data limitations. Here, we provide a novel method to map slaughterhouse expansion and contraction. We analyzed the opening and closing of 133 plants between 1967 and 2016 in Mato Grosso and estimated the geographic locations and slaughter volumes. This was achieved by triangulating across multiple data sources including a registry of 21 million companies, government records of three million slaughter transactions (Portuguese acronym GTA), and high resolution satellite imagery. Our study is the first to include longitudinal information and both inspected (for food quality) and uninspected slaughterhouses. The results show that 72 plants operated in 2016 through 52 holding companies. By measuring geographic distances between active plants and pasture areas, we documented a 29% increase in the density of plants during 2000–2016, showing an expansion of the cattle slaughter infrastructure. We identified three periods of expansion: 1967–1995, with 15.1% of the plant openings; 1996–2003, with 24.6%; and 2004–2016, with 60.3%. While closings likely occurred throughout the period studied, no data were available prior to 2002. We estimated a minimum value for the volume of uninspected slaughter as 2–3% for 2013–2016. We conclude by discussing potential applications of the data, a deidentified version of which is made available through an online repository. The method developed here can be replicated for the whole country, which would increase our understanding of the dynamics of cattle slaughter and their impact on land use.

## Introduction

The rapid improvement in our capacity to map the world through big data and satellite imagery has powerful implications for policy as well as for business strategy. The agrifood industry can now track the source of their inputs at the scale of a single box by using radio frequency identification technology [1]. Farms can optimize fertilizer and pesticide use through smart farming [2] and satellite-based precision agriculture [3]. Retailers can use satellite images to measure client flows by counting cars in parking lots [4]. Non-governmental organizations and governments can target poor households for social interventions [5] or improve their response to humanitarian crises by using high-resolution satellite images [6]. The examples are plentiful, and the full potential of the new data sources is far from explored.

In parts of the developing world where rural areas are changing rapidly, such as the Brazilian Cerrado and Amazon biomes, or the Gran Chaco region in South America, documenting the evolution of new forms of land use is essential for timely policy interventions [7, 8]. However, the necessary data are often either unavailable or stored in databases that were designed for disparate purposes. Here, we explore a case where the careful triangulation of public information sources along with the use of open-access high-resolution satellite imagery greatly improved our ability to map supply chains. The beef industry in Brazil has a substantial economic and environmental impact, yet knowledge of its dynamics remains incomplete, particularly with regard to slaughterhouses. By focusing on Mato Grosso–a Brazilian state the size of France and Germany put together and a cattle ranching powerhouse that spans the Amazon and Cerrado biomes–we developed a new method to map the slaughter industry across space and time.

The modern conception of slaughterhouses dates back to the early 19^th^ century, when the transition from an agrarian to an urban-industrial system created the demand for cleanliness, less visibility of the slaughter of animals, and more efficiency: “[i]*n 1807*, *Napoleon ordered the building of public abattoirs to provide meat for Paris*, *and then for other French cities*” [9]. As slaughter facilities evolved from pre-industrial abattoirs to large-scale meatpacking enterprises (of which Chicago’s Union Stock Yard, founded in 1865 and closed in 1971 after the U.S. meatpacking industry decentralized, is a well-known example), the industry moved from a butchery-style business to a more factory-like standard [10].

Documenting the slaughter industry dynamics in the Amazon is important for economic, conservation, and sanitation policies. Mapping the expansion of the slaughter infrastructure will improve our understanding of the patterns, drivers, and impacts of increased agglomeration and market power by larger players [11–15] and the spatial distribution of the industry’s installed capacity [16], which affects the welfare of both cattle producers and beef consumers. In terms of agricultural and conservation policy, understanding the role of infrastructure development on the dynamics of land cover change is important [17, 18]. While a key predictor of the expansion of cattle-related deforestation is the provision of roads [18, 19], the role of slaughterhouses, a central type of infrastructure, remains elusive. Do slaughterhouses precede the expansion of cattle ranching, or the opposite? What drives company decisions for locating new plants and how does that vary through time and space?

Another benefit of generating such data is to produce a more precise understanding of the market share, distribution, and time dynamics of uninspected slaughter. This can be a central input to policymaking in the food inspection arena. The slaughter of animals with poor sanitation standards is a serious concern in the poorer areas of Brazil. In the state of Amazonas, for example, which in 2016 had four million inhabitants, 71% of the installed slaughter units were not subject to any sanitation inspection [20], implying that a substantial share of the beef products consumed in the state had questionable food quality standards. For Brazil as a whole, the estimated rate of uninspected slaughter is between 10.7% and 8.9% [21, 22], where the Brazilian Statistical Agency (IBGE) calculates the difference between the reported count of cattle hides and the slaughter reported in inspected units, while CEPEA (Center for Advanced Studies in Applied Economics) subtracts the reported inspected slaughter from modelled estimates of the total beef consumption. Yet targeted action by sanitation agencies requires information on the location and capacity of the uninspected plants, which is currently unavailable.

Knowledge of the location and clustering patterns of different types of animal slaughter infrastructure, and of ownership structure and changes, has been constrained by severe data limitations. Not surprisingly, the best public data currently available provide only cross-sectional snapshots of the larger plants [23, 24], which limits analysis and policymaking efforts. Applications involving distant locations and markets where the slaughter infrastructure is mostly small scale are especially hindered by the lack of data.

In this paper, we introduce the first longitudinal assessment of the cattle slaughter industry in Brazil. After this introduction, we provide background information on the industry and the limitations of the existing data. Next, we describe the study area and present the paper’s methodological procedures. In the methods section, we first provide a step-by-step explanation of how we mapped the space-time signature of slaughterhouses; second, we present the data validation procedures; and third, we describe how we analyze the results to learn about the temporal dynamics of slaughterhouses, pastures and cattle. The results section provides information on the location, temporal dynamics, and slaughter volume of the slaughterhouses. Based on that, we provide estimates of the minimum size and distribution of the uninspected market. We also present the data validation results. Finally, we generate maps of the expansion of slaughterhouses across time and space and compare their evolution to the dynamics of grazing areas and cattle herds between 2000 and 2016. In the final section, we discuss the results and data limitations, list potential applications of the data, and provide new insights for the study of cattle and land use change.

## Background and existing data

Slaughterhouses have a central role in organizing supply chains, exerting influence on the size, type, and location of ranches [10, 25, 26]. In Brazil, cattle herds and abattoirs have expanded since the late 16^th^ century [26] as cattle, buffalos, sheep and other bovid animals were essential for food, clothing and transportation in colonial times [27]. The movements of humans and herds toward the hinterland were intrinsically connected throughout Brazil’s history [26]. In Mato Grosso, cattle were present since at least 1720 in Cuiabá, while the internal demand for beef saw its first surge circa 1750, with the building of the road to Vila Boa de Goiás, in today’s state of Goiás [28]. Such coevolving pattern of human settlements and cattle herds continues, especially in the frontier regions of the Amazon and Cerrado biomes [29, 30]. Accordingly, the infrastructure necessary to slaughter animals remains a constraint for the expansion of human settlements.

As of the last four decades of the 20^th^ century, bovine herds saw a steady movement toward the Amazon region [31], spurring environmental concerns due to the land-intensive nature of the activity. The most recent wave of expansion has turned Mato Grosso into Brazil’s leading state for cattle production with 13.9% of the country’s 218.23 million head [32]. Pará and Rondônia, states that are fully inside the Amazon biome, also saw their herds grow by 141% and 199% in sixteen years, becoming the 5^th^ and 6^th^ largest herds. In this recent expansion wave, slaughterhouses became even more important players in the supply chain, increasingly influencing production practices at the farm level [33–35].

Information about the temporal and spatial dynamics of the slaughter infrastructure in Brazil is limited. Only recently have comprehensive maps of the larger slaughterhouses become available [23–24], but they provide only a contemporary snapshot of the larger plants. When were the plants created? When did holding companies merge? When were plants deactivated or closed? What were the existing plants at each point in time, and what was their productive capacity? Knowledge of the local level infrastructure is also weak, with a gap in the understanding of the volume of slaughter in facilities with different types of food safety inspection.

Slaughterhouses in Brazil are classified according to their sanitation inspection status: federal, state, municipality-level inspection, and uninspected. Those under federal inspection are attributed a SIF (‘Federal Inspection System’) code and can sell meat products anywhere in Brazil, or abroad subject to a special license. Plants with state inspection (SIE) have within-state market access, while plants with municipality inspection (SIM) are restricted to the county. All SIF, SIE, and SIM plants should, in theory, be inspected by a veterinarian to ensure the health of the animals. Uninspected plants tend to be small, local abattoirs and are more common where there is a less developed institutional framework. For example, the poorer states in the Northeast of Brazil as well as remote locations in the Amazon, where distances are large and population densities low, have a higher frequency of uninspected facilities [20, 22].

Unmet sanitation standards can have serious consequences. Health implications for humans include the direct contamination by bacteria such as Salmonella and Escherichia Coli, Brucellosis (a contagious zoonosis), Taeniasis (infection with Taeinia tapeworms), and Toxoplamosis–all of which can lead to death–but also the increased chance of environment-related contaminations due to the improper management of waste products. Poor sanitation at slaughter can also send a signal to livestock producers that animals without appropriate vaccination and health care are acceptable. This can lead to an increased risk of epidemiologic events such as the spread of the highly contagious viral foot-and-mouth disease. Finally, the economic consequences include a higher likelihood of low labor standards as well as depressed prices due to lack of market access.

In 2016, the Ministry of Agriculture estimated that 10% of the beef products coming out of SIF plants were in breach of minimum sanitation standards [36]. Whereas IBGE shows that uninspected slaughter in Brazil is on a downward trend, poorer areas lag behind. Estimates by [10] suggest that in São Paulo the share was 5.4%, but in the Northeast it was 10.4% and in the North region as much as 11.2% of all cattle commercially slaughtered were uninspected. Moreover, even if most slaughter is sanitation-inspected, inadequate inspection remains an issue. The federally inspected plants are recognized as the most likely to comply with sanitation standards. For municipality- and state-inspected units, a thorough 2013 assessment concluded that 80% of the plants across the country were out of compliance [37]. Lack of inspection and inadequate inspection are serious problems.

One part of the uninspected market remains obscure despite all data gathering efforts. Clandestine slaughter is an illegal activity that takes place without a formal business registration, so it is absent from all official records. This includes slaughter for auto-consumption taking place sporadically inside farms and ranches, but also unregistered abattoirs of different sizes. It is possible for a company to have a legal business registration (‘National Cadaster of Legal Persons’, CNPJ) while not being inspected for food quality, but clandestine slaughter is the lack of both a registration and inspection. Uninspected slaughter is thus a general concept that is more easily quantifiable as it can take place with a formal business registration.

For the clandestine market share, only indirect inferences can be made. One assessment about its size estimated a market share between 26% and 67% in 1996 across Brazil [38], but it made no distinction between clandestine and uninspected slaughter. Another study by [33] estimated a 21% market share in 2009 by comparing the total number of hides processed to the total slaughter volume. By 2016, this share had dropped to 13.2% [39, 40], but the method also lumps together uninspected with clandestine slaughter. Finally, a minimum value for the clandestine share in Brazil can be inferred from CEPEA’s estimate of auto-consumption (in-farm slaughter) of 10% in 2012 [22]. In terms of its spatial distribution, the best that can be said–given the data limitations–is that the prevalence of clandestine slaughter is likely higher in less developed locations, where regulations are less enforced, and that locations with a greater incidence of uninspected slaughter may also have more clandestine facilities.

## Methods

This study involved a limited amount of field research that was approved by the University of Wisconsin Madison’s Education and Social/Behavioral Sciences Institutional Review Board under study number 20130044 (principal investigator: Holly Gibbs).

### Study area

Mato Grosso is the 3^rd^ largest state in Brazil (903,357 km^2^) and a key conservation target for the Amazon and Cerrado biomes, which respectively occupy 54.5% and 38.3% of the state’s territory (the Pantanal wetlands cover 7.2%). It has been leading the modernization of Brazilian agriculture and cattle ranching for over a decade, especially in large-scale commercial agriculture [41–43]. Mato Grosso has the largest share of agriculture in its Gross Domestic Product (GDP) among all states, 21% in 2014, and the 5th largest agricultural GDP [44]. The expansion of mechanized agriculture, especially soy, has been pointed out as one reason for the state’s development process [45]. For example, Mato Grosso had the largest GDP growth relative to the country in 2002–2014, and the fourth largest per capita income growth in the same period [44, 46].

With a small population (1.6% of Brazil’s total) but the largest cattle herd (13.9%), Mato Grosso has a dynamic slaughter industry that supplies to other states and overseas. It is the second biggest beef exporter after São Paulo [47], and only 18% of its production is consumed in-state [48]. An important part of the industrial agglomeration that took place in Brazil since approximately 2005, which led to the creation of the largest meatpacking conglomerate in the world [49], involved operations in the state of Mato Grosso [50].

Land use patterns in Mato Grosso are atypical for Brazilian standards. Pasture area growth decelerated more rapidly than in other locations, while the opposite happened to crop areas. According to the agricultural census, pastures in Mato Grosso grew by 38.3% per decade in 1975–1995, and by 45.6% in other states in the Legal Amazon. In 1995–2006, however, Mato Grosso had an additional 2.8% pasture area, while the other Amazon states had a 31.8% increment [51]. The total crop area, on the other hand, grew by 117.7% in 1995–2006 [51, 52], and by 116.1% in 2001–2014 [53]. In more recent years, while census data are unavailable, remote sensing data show that pasture areas continued expanding by a very small percentage [54, 55]. This has come with increased cattle densities, as we show in the Results section.

### Mapping the space-time signature of slaughterhouses in Mato Grosso

‘Slaughterhouse’ is a generic term that may refer to a physical plant or to a holding company depending on the context, so we start by clarifying the definitions. ‘Plants’ or ‘units’ are physical instances of individual slaughtering operations. In this study we chose to focus on plants that slaughter at least 300 head per year, which may be called abattoirs but not butcheries. This choice was made because very small facilities have a negligible impact on quantities but demand more data processing work as data are either unavailable or less transparent. Hence, where plants with slaughter volumes of <300 head appeared in the dataset without an address, we made no effort to retrieve the address from additional sources and instead grouped those CNPJs together into one ‘unidentified’ plant per municipality.

‘Holdings’ are the companies that own the physical plants. Each plant has a single holding company at each point in time, but multiple plants can be owned by a holding. We documented and report only the most recent holding company of each plant, and as such miss the dynamics of previous ownership changes. Our definition of holding comprises local businesses that own at least one small plant. ‘Legal persons’ are the formal business registrations that the Federal Revenue agency issues to companies in Brazil. These legal identities, known by the acronym CNPJ, are used to process corporate taxes and financial information. One plant will sometimes operate through two or more CNPJs.

**Fig 1:**
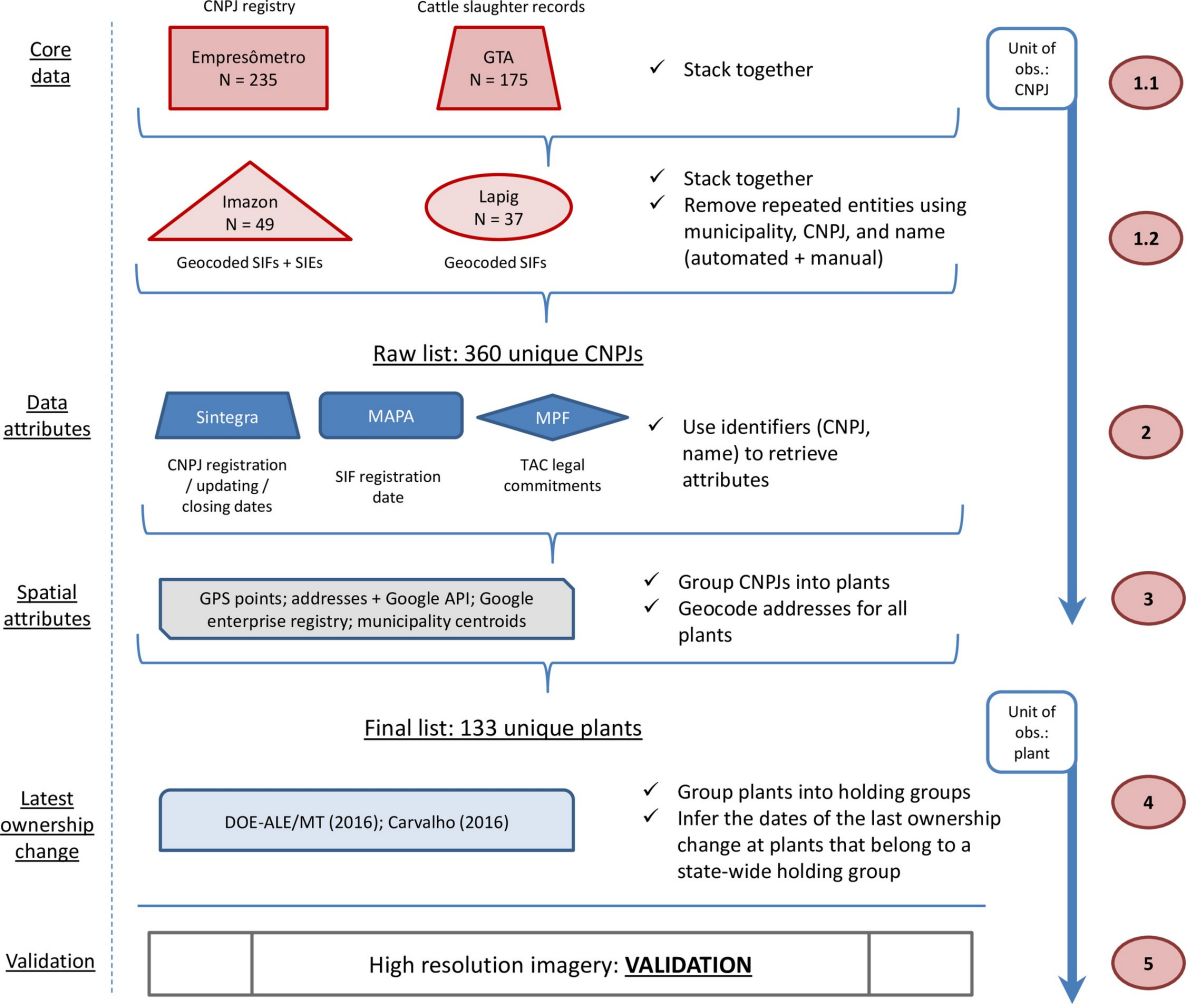
Methods flow chart. SIFs/SIEs are slaughterhouses with federal/state inspection. See S1 Table for definitions of Empresômetro, GTA, Imazon, LAPIG, Sintegra, MAPA, and MPF.

To generate a map of all slaughterhouses in Mato Grosso, we triangulated across multiple data sources including a registry of companies, government records of cattle transactions and of the sanitation inspection system, data compilations by a think-tank and a research lab, open-access high-resolution satellite imagery, and others (S1 Table). Fig 1 provides a schematic view of the data sources used and the sequence of steps applied. The process was divided into five steps that we describe below: compiling a core dataset with key company identifiers, such as names and addresses; populating the dataset with attributes from multiple sources, especially opening and closing dates (of a CNPJ); grouping registered companies by physical plant and geocoding the addresses; documenting and inferring ownership changes and dates for the larger holding groups; and validating the data through comparisons with other sources of information.

We started by stacking up the two main sources of raw data (step 1). The first is a Brazil-wide company registry compiled by Empresômetro using data from the Federal Revenue agency on CNPJs and their names, legal names, economic activities, dates of creation, and addresses. We filtered 21 million records to obtain a list of 235 businesses registered across Mato Grosso under economic activities related to cattle slaughter. We used CNAE codes, an official classification of economic activities, to select CNPJs registered as slaughterhouses. One (or more) CNAE code is assigned to every CNPJ. We selected all CNPJs with at least one of the following CNAE codes: cattle slaughterhouses (code 1011201) and cattle abattoirs (code 1011205), or with at least one of the following words: “frigorífico” (slaughterhouse), “matadouro” (abattoir), “carne” (meat), and “abate” (slaughter). The second source is a compilation of government records (GTA, the Portuguese acronym for ‘Animal Transportation Form’) on cattle slaughters from Indea-MT, the state livestock sanitation agency (S1 Box). The GTA records include 2,976,962 transactions, from which we identified 175 companies (slaughterhouses or abattoirs) responsible for slaughtering cattle between 2013 and 2016.

Next, we added the data from Imazon, a Brazilian environmental think-tank, and from Lapig, a remote sensing and geoprocessing research lab. The Imazon data include inspection codes and the geolocations of 49 plants under federal or state inspection, and the Lapig data have the same information for 37 plants with federal inspection. We dropped observations with repeated CNPJs to get a raw list of 360 registered companies.

In step 2, we populated the dataset with the attributes in S1 Table. We used three sources of information: Sintegra, a web gateway to information from the State Revenue agencies; MAPA, the Brazilian Ministry of Agriculture; and MPF, the Federal Prosecutors Office. Sintegra can be queried using a CNPJ number and it provides the most accurate opening and closing dates. The MAPA portal allows for queries by SIF code, and it is the only source for start date of inspection at SIF plants. The MPF portal is the only source for the signing dates of the TAC (Portuguese acronym for ‘Conduct Adjustment Term’) supply chain commitments–binding contracts signed between slaughterhouses and Federal Prosecutors for properties in lack of compliance with environmental/labor standards to be excluded from supply chains [34]–and it provides a list that we matched to our database using names and municipalities.

At this point, the units of observation were still the CNPJs, but these do not bear a one-to-one relationship with physical plants. Due to fiscal or otherwise managerial motivations, an active slaughterhouse plant operates, on average, through between one and five active CNPJs [mean = 1.5]. In step 3, we aggregated the CNPJs into plants. While many CNPJs can be associated with one plant, multiple plants were never associated with a single CNPJ in our dataset. We grouped CNPJs within plants only if they were registered in the same municipality. All CNPJs with name strings and/or addresses that we judged to be the same were given a unique plant identifier. All CNPJs for which no company name or address information was found at a single municipality were placed under one ‘unidentified’ plant. The final dataset has 133 plants.

For the spatial coordinates, we followed a hierarchical decision rule. First, we used GPS points obtained in field visits. Second, we used the coordinates provided by Google on its enterprise registry. Third, we used the coordinates provided by [23, 24], both of which were visually inspected using high resolution satellite imagery. Fourth, we used Google Maps to geocode the addresses provided in the company’s CNPJ registry.

Similar to plants operating through multiple CNPJs, a holding group may control multiple plants across the state. In step 4, we grouped plants into holding companies. Plants with at least one CNPJ whose name or legal name was that of a known holding group, defined as any group listed in [23], the most comprehensive list available, were allocated to the known holding. If the name of a known holding group did not appear in any of the plant’s CNPJs, we chose the shortest name between all CNPJs as the holding name. In less than 5% of the cases, one plant had CNPJs referring to different known holding groups, so we used expert consultation, online news portals and other local sources of information to identify the holding controlling the plant at present.

After the plants were aggregated into holdings, all the plants within each holding were coded as having signed a TAC commitment when at least one CNPJs associated to any of the plants within the holding had a TAC in place.

Where a plant from a known holding group had CNPJs with different names, the plant was flagged as having gone through an ownership change. We used [48, 50] to document the timing of the last ownership change. Where the date was not documented in those sources, we used two alternative assumptions. First, when a holding buys a new plant, it will often create a new CNPJ and will discontinue one or more CNPJs under the previous name. We used a switch in CNPJs as an indicator of ownership change. If there was no temporal coincidence between the closing and the opening of CNPJs, then we took the opening date of the most recent CNPJ under the current holding’s name as the ownership change date.

Finally, in step 5 we validated the spatial coordinates by the visual inspection of high resolution imagery on Google Earth. We assessed whether the mapped location showed the typical structure of a slaughter facility, which includes cattle corrals, industrial buildings and waste storage lagoons. High resolution images are available for relatively recent years, so plants that closed before the early 2000s were more difficult to identify. Our data have one closing date earlier than 2005 and 20 earlier than 2010 (33% of the closed plants). This was a relatively minor problem, however, because even the plants that closed before satellite images were available could in some cases be identified since their structure remained visible years after the closing.

#### Inference of plant activity and inactivity

We used the company’s opening and closing dates along with the dates of the GTA records to establish whether a plant was active or inactive (these terms are used interchangeably with open / closed) at a given year. Activity is defined as an ongoing company registration (at least one open CNPJ) for the years when no GTA information is available (prior to 2013) or positive slaughter activity for other years. For example, if there was at least one active CNPJ but no slaughter activity in 2014 and after, the plant was coded as inactive in 2014 and after. If there was slaughter activity in 2014 but no legally active CNPJ, the plant was active in 2014. If there was at least one active CNPJ in 2007, the plant was active then. If there was no recorded slaughter activity in any year, the company was coded as inactive in the latest year when one of its CNPJs became inactive, even if one or more active CNPJs remained. In seven situations–which we coded as active–did a plant show a positive slaughter activity in years subsequent to the closing of its last CNPJ (94 plants had GTA slaughter activity).

CNPJ closing dates were taken from two sources. The company registry, which shows if the company registration was discontinued at the federal level, and Sintegra, that shows whether the company’s state fiscal registry was discontinued. If at least one of these sources showed a closing date, we coded the CNPJ as closed.

The opening date was defined as the earliest date between the SIF registration date and the earliest CNPJ registration date. The Ministry of Agriculture’s data on when SIFs were first registered is the most accurate historic information for the decades of the 1970s through the 1990s. For non-SIF slaughterhouses, we used only the company registry. For plants operating earlier than 2013, these are the only sources of information for the starting date as the public GTA slaughter records start in 2013. For plants that were operative in 2013 or after, the GTAs allowed us to estimate the starting date even if the plants had no legal registration or sanitation inspection. In these cases, we assigned the first appearance in the GTA as the opening year.

#### Estimating uninspected slaughter

The weakest part of the animal inspection system is the municipality [56]. According to [37], only 20% of SIM or SIE plants were compliant with sanitation standards in 2012. Assuming that SIMs are even less likely to be compliant than SIEs, it follows that SIM plants are unlikely to be fully inspected. This is the first reason why we code SIM plants as uninspected. The second reason is that, even if desirable, distinguishing SIMs from uninspected at a large scale is not possible due to the lack of consistent data on the location of SIM plants. So for simplicity, we refer to both as *uninspected*. In the past, even the plants with state inspection where classified as uninspected due to the lack of data [38]. Moreover, only seven plants in Mato Grosso were reported by IBGE as being under municipality inspection in 2016 [39]. Considering the 20% compliance rate, this could imply that two of the seven SIM plants in Mato Grosso are effectively inspected.

We used the following formula to calculate the slaughter volumes of uninspected plants at the municipality level:

$${Tot\_SL}_{tm}={SIF\_SL}_{tm}+{SIE\_SL}_{tm}+{UN\_SL}_{tm},$$ (1)

Where the subscripts *t* and *m* indicate year and municipality, *Tot_SL* is the total slaughter volume, *SIF_SL* is the slaughter in plants inspected by the federal authorities, *SIE_SL* is the slaughter in plants inspected by the states, and *UN_SL* is the estimated uninspected slaughter (including clandestine and non-clandestine). By using the GTA slaughter records we have largely captured the non-clandestine market, as all GTA transactions must include a CNPJ number. The only GTA transactions that could refer to clandestine plants are those whose CNPJs do not match with any known CNPJ record, which amounts to only 1.74% of all the transactions in our data.

### Data validation

We used external sources of information (other than those presented in S1 Table) to validate our results and discuss uncertainty and error in the data. First, we assessed the degree to which the sample used is representative of the population of slaughter transactions. We compared the total head slaughtered in the GTA to data from IBGE [39]. The IBGE records are collected from informants in the slaughterhouses, while the GTA records are collected from the ranchers selling to the slaughterhouses.

Second, we evaluated the historical accuracy of the data. Registration years indicate the formal registration of companies, but the plants may have operated from an earlier time or the registration date of those that were closed before the digital era may have gotten lost. For this, we conducted expert consultations with a senior veterinarian from Mato Grosso’s sanitation system and with four cattle ranchers who moved to the Alta Floresta region in 1976. We thus collected historic information on the first slaughterhouses operating in parts of Mato Grosso.

Finally, to validate our estimates of the uninspected market, we used municipality-level data on formal employment from the Brazilian Ministry of Labor. The employment data provide counts of workers in businesses registered as bovine cattle slaughter units across the country. If a municipality does not have any inspected slaughterhouse but does have workers registered in the slaughtering industry, then it is likely to have uninspected cattle slaughter.

### Analyzing the dynamics of slaughterhouses, pasture and cattle over time

To put the evolution of slaughterhouses into context, we produced maps of their expansion as compared to grazing areas and cattle herds for the years 2000–2016. For cattle herds, we used municipality-level yearly data from [33] to calculate cattle densities for each year and municipality. For pastures, we combined [54] and Lapig [56] pasture classifications to produce maps of the maximum area under pasture at each year. The Lapig product classifies pasture and non-pasture based on Landsat-8 images with a resolution of 30 meters. The Mapbiomas product uses Landsat 5, 7 and 8 with a 30 meter resolution. We coded the areas classified as “pastures” or “pastures or agriculture” as pastures. We then overlaid all pasture maps to obtain a maximum pasture area among all years. From that, we sampled 15,000 random points, in three iterations, to represent the maximum pasture surface of Mato Grosso (S1 Fig).

For each year, and for all the points classified as pasture, we retrieved cattle densities (head/ha) and the average Euclidean distance to the three nearest active slaughterhouses. We did this for the three iterations. The distance to the slaughterhouses is a proxy for the density of plants with regard to pastures, so an increasing number of slaughterhouses in a slow-increasing pasture surface yields a decreasing average distance. We then computed yearly cattle densities and distances for the state by averaging among the points classified as pasture at each year.

## Results

### Expansion and contraction of slaughterhouses

**Fig 2:**
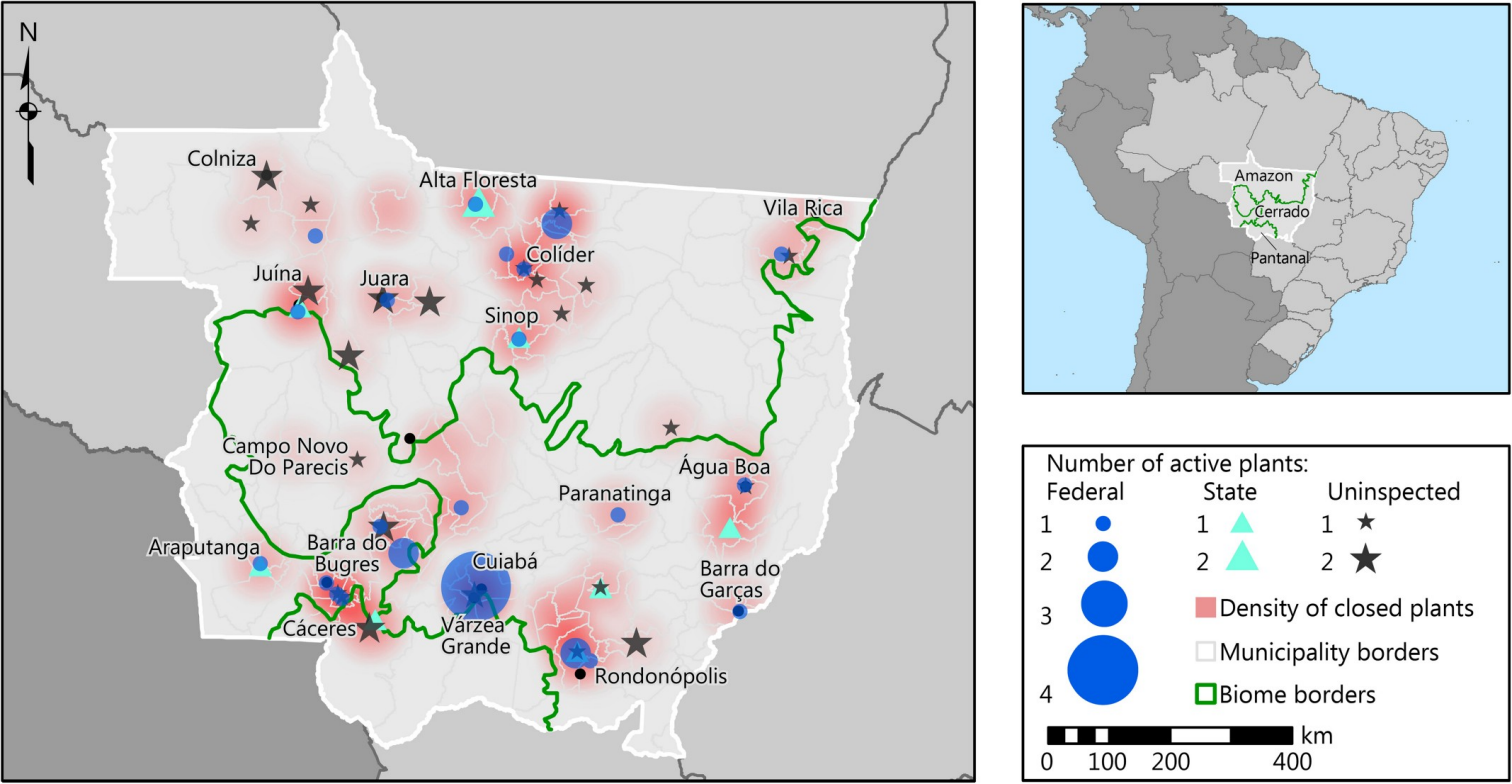
Study area, active slaughterhouses (2016) and clusters of closed plants (2002–2016). The red clouds are heat areas estimated from kernel densities of plants closed (all years) over a radius of 50 km around each plant location; darker red indicates locations with a higher historical frequency of plant closings. Most areas with closings are also where there are active plants today, except for the area just north of Cuiabá, where soy has largely replaced pastures, and an area west of Alta Floresta. A plant was classified as active if it had positive slaughter activity in 2016. All plants without federal (SIF) or state (SIE) inspection were classified as uninspected. For seven plants with a closing date but without a starting date, we estimated the starting date using the average life-cycle of the plants in the same inspection category. Sources: [23, 24, 58]; company registry (CNPJ), Empresômetro; Sintegra; Taxpayer Central Registry; Ministry of Agriculture (see S1 Table for more details on sources).

A deidentified version of the data resulting from this paper is available at [57]. This section presents and discusses the evolution of the slaughter industry, making reference to the historical dynamics of cattle ranching in Mato Grosso. Active plants are mapped in Fig 2 by inspection system for 2016, the last year for which GTA records are available. A total of 30 SIF plants are distributed across the state. The Várzea Grande area around the capital has the highest concentration (four plants), while the northeast (Colniza) and northwest (Vila Rica) regions have the lowest SIF concentrations. Plants with state inspection are a minority–a total of nine–although they account for 8% of the slaughter volume (Table 1). A maximum count of 11 SIE plants was reached in 2013.

The SIF plants have a wide and stable dominance of the market in Mato Grosso–around 90% of the slaughter volume (Table 1). The large market share of SIFs is not exclusive of Mato Grosso: Rondônia, Tocantins, Mato Grosso do Sul and Goiás have similar situations [59]. In Mato Grosso, the group of three meatpackers known as G4 (JBS, Marfrig, and Minerva) has accounted for the bulk of the SIF share, although the G4 share has fallen from 71% to 66% of the total slaughter volume (2013–2016). This is due to a shrinking market share for JBS combined with a growing market share for non-G4 SIF slaughterhouses. Contrary to this movement, the TAC signatory SIF plants saw a slight increase in their market share (from 80% to 83%). Overall, zero-deforestation and anti-forced-labor policies have a wide coverage in Mato Grosso.

**Table 1:** Slaughter volume shares in Mato Grosso by inspection system, 2013–2016.

Year	2013	2014	2015	2016
	Head slaughtered in Mato Grosso			
Total GTA*	5,756,329	5,317,795	4,482,383	4,411,081
SIF plants^1^	91%	91%	90%	89%
G4	71%	68%	67%	66%
TAC	80%	83%	82%	83%
SIE plants^1^	5%	6%	7%	8%
TAC^2^	3%	4%	5%	5%
Uninspected^3^	3%	2%	3%	3%
Total^4^	5,837,857	5,352,226	4,540,805	4,577,459
SIF plants	94%	93%	91%	91%
SIE plants	6%	7%	8%	9%
SIM plants	0.2%	0.4%	0.5%	0.5%

The substantial drop in slaughter volume after 2013 (> 20%) was in large part due to the economic crisis that hit the whole country after 2013.

*Excludes head finished in other states and slaughtered in Mato Grosso.

^1^Our data do not include information on historical inspection status. Therefore, we assume that the inspection status in all years in which a slaughterhouse was active prior to 2016 was the same as in 2016.

^2^All slaughterhouses under TAC are assumed to have been under TAC since 2013.

^3^All slaughterhouses that do not currently have a federal inspection (SIF) or state inspection (SIE) code are treated as uninspected at all years

^4^[49].

Sources: same as in Fig 2.

**Fig 3:**
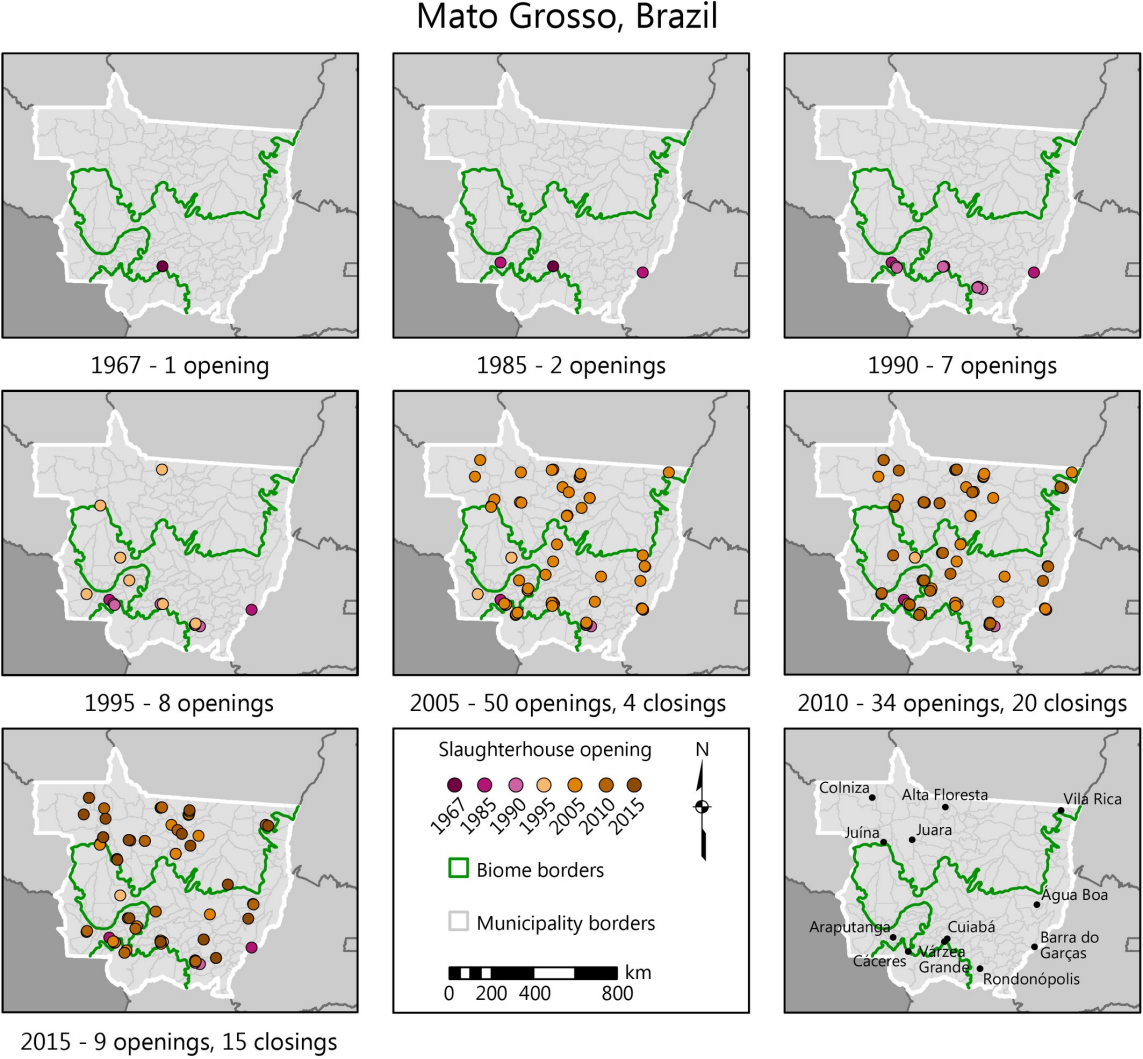
Slaughterhouses in Mato Grosso by starting year, 1967–2015. Circles represent individual plants. The opening (closing) date is inferred from the company registry where available, from the earliest (latest) slaughter transaction date, or from the Ministry of Agriculture’s records. For years before 2013, only companies that were formally registered as slaughterhouses are accounted for, so the evolution from 1967 to 2013 indicates both actual births and deaths of slaughterhouses and the increased formalization of companies. From 2013, all slaughter transactions recorded at the state’s sanitation agency are mapped. For seven plants with a closing date but without a starting date, we estimated the starting date using the average life-cycle of the plants in the same inspection category. Sources: same as in Fig 2.

The first slaughterhouse registration that we could map appeared in 1967 near the state capital, Cuiabá (Fig 3, top-left panel). The second and third plants opened in the eastern location of Barra do Garças, and in the municipality of Araputanga, at the border between the Amazon and Pantanal biomes (Fig 3). The southwestern region of Cáceres, where Araputanga is located, is the oldest post-colonial settlement of Mato Grosso and one of the main routes through which cattle penetrated from the Pantanal after the downfall of the extractive industry in the early 20^th^ century [28]. A few years later, another slaughterhouse was registered in the neighboring municipality of São José dos Quatro Marcos. Not surprisingly, the southwest is one of today’s strongest clusters of the cattle ranching industry in the state.

**Fig 4:**
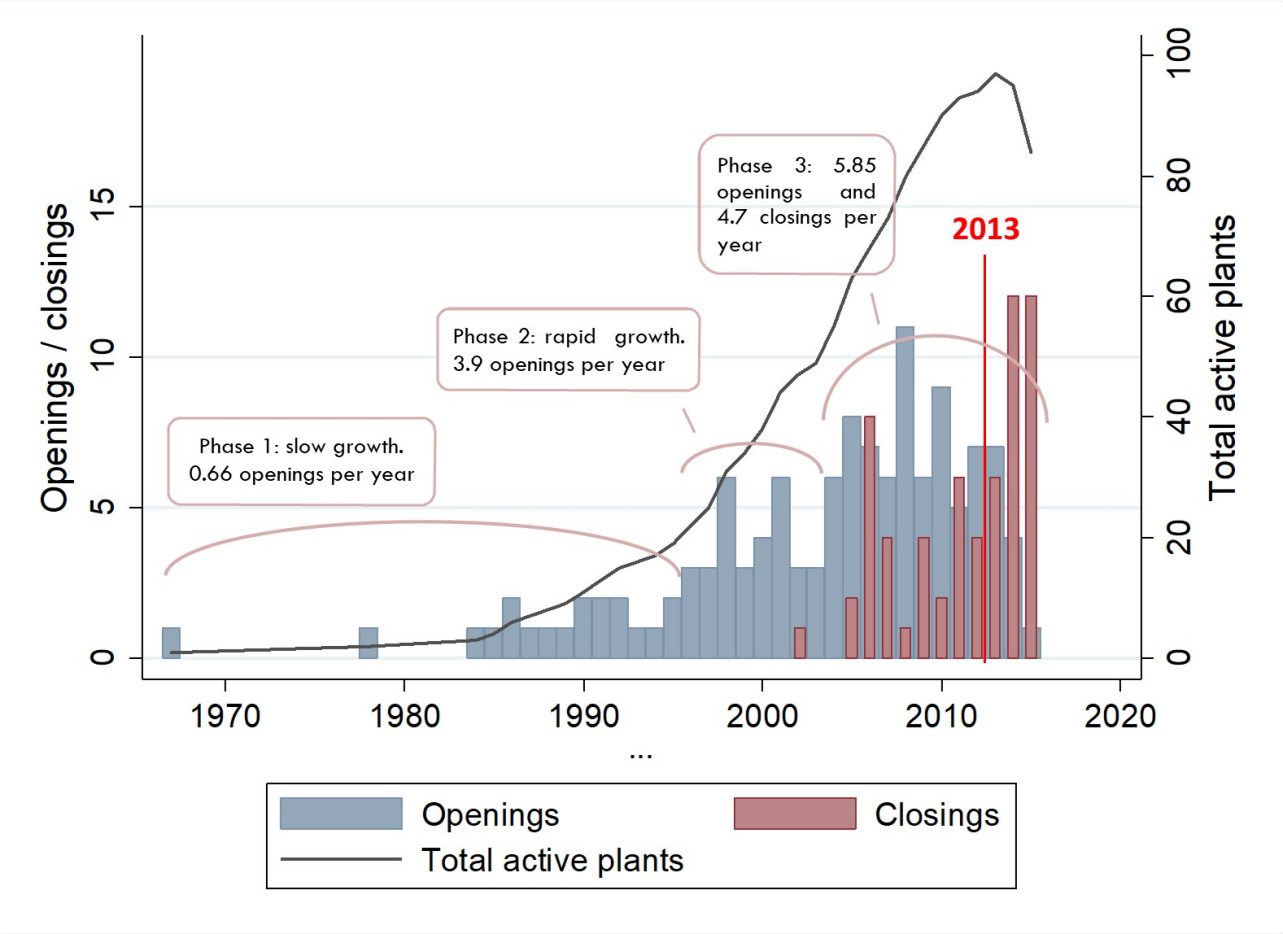
Count of estimated openings and closings of slaughterhouses, 1967–2016. The opening (closing) date is inferred from the company registry where available, from the earliest (latest) slaughter transaction date, or from the Ministry of Agriculture’s records. For years before 2013, only companies that were formally registered as slaughterhouses are accounted for, so the evolution from 1967 to 2013 indicates both actual births and deaths of slaughterhouses and the increased formalization of companies. From 2013, all slaughter transactions recorded at the state’s sanitation agency are mapped. Seven plants that have closing dates in 2006 but no opening dates are accounted for in the closings but not in the total active plants. Sources: same as in Fig 2.

Rondonópolis, in the southeast, is the second largest city in the state and a key cattle area located at the major axis that connects Mato Grosso to the older cattle states of Mato Grosso do Sul and Goiás. The first slaughterhouse registrations in Rondonópolis are from 1987 and 1988. Other plants were registered across western Mato Grosso at the rate of 1–2 per year until the mid-1990s (Fig 4). By 1998, the population of registered slaughterhouses covered most of the areas that are now strongholds of the meatpacking industry: the southwestern triangle between Cáceres, Barra do Bugres and Cuiabá; the southeastern triangle between Rondonópolis, Barra do Garças and Paranatinga; the north around Sinop and Colíder; and the northwest around Juína.

The registration of new plants only took off in 1996 (Fig 4). From then until 2015, a discernible new trend emerged. The average rate of slaughterhouse openings was 3.9 per year until 2004, then 5.85 per year until 2016, with a solid expansion that lasted 12 years. (Caveat: Companies that appear only in the GTA, and that do not find a match to the company registry, are recorded for the first time in our database in 2013 or after. Since the GTA slaughter records go as far back as 2013, our estimates likely overshoot the year of initial operation for some companies. As a consequence, the pre-2013 count of plant births is likely underestimated, while the post-2013 count is overestimated.) Also, around 2011 a pattern emerged where the number of closings was equal to or higher than the number of openings, signaling that the rapid expansion period had found its limits.

The period between 1996 and 2012 saw rapid change. The Brazilian currency was devalued in December 1998, creating a stimulus for exports that was one of the drivers for the expansion of deforestation, which peaked in 2004 [60]. Mato Grosso obtained the status of foot-and-mouth disease-free state in 2000 [61], spurring the installation of export-oriented plants. The rate of business formalization was accelerated by stronger fiscal enforcement as well as new legislation that facilitated the registration of companies (Law 123, Dec. 2006). The zero-deforestation cattle agreements began in 2009 following a campaign by Greenpeace and in the years that followed with the Federal Prosecutors office [34]. Moreover, the meatpacker industry became increasingly concentrated in the hands of large players such as JBS, Marfrig, and BR Foods [48].

Fig 4 shows that by 2016 many plants had gone through a full life cycle. We identified 61 slaughterhouses that opened and closed in Mato Grosso since 1967, the majority of which were small, uninspected units (S2 Table). The locations where most of the closings took place correspond to where the first slaughterhouses appeared: Araputanga, Cáceres, Rondonópolis, Barra do Garças, and more recently the Alta Floresta-Sinop axis in the north (Fig 2). The exception is Várzea Grande, the municipality with the highest slaughterhouse density in the state, where only two out of eight plants have closed. From the 72 active plants in 2016, the oldest ones were the SIFs, followed by SIEs and uninspected plants.

#### Uninspected slaughter

Uninspected plants are also relatively well distributed throughout the state, with a high prevalence in the north. While their market share is low (2–3%), these plants serve the local markets and impose few restrictions on suppliers. This implies that areas with high deforestation and significant cattle expansion, such as the northwestern region of Colniza, are more likely to have uninspected units. Indeed, the map shows that the northwestern corner has a low prevalence of SIF or SIE plants. The extreme northeast, another area of significant recent cattle expansion, had no SIF slaughterhouse until 2010 [62] and is now served by a combination of SIF and uninspected plants.

The life-cycle of uninspected plants is about half that of inspected plants (S2 Table). Because of the shorter life span, there are almost as many closed uninspected plants as there are active ones. Larger plants, on the other hand, tend to suspend activities or be incorporated into other holding groups instead of closing their operations. Because of this, they display a higher CNPJ turnover (as CNPJs that belonged to previous owners are discontinued). SIE plants operate with almost three active CNPJs, on average, suggesting that there may be benefits to using more than one legal persona.

**Fig 5:**
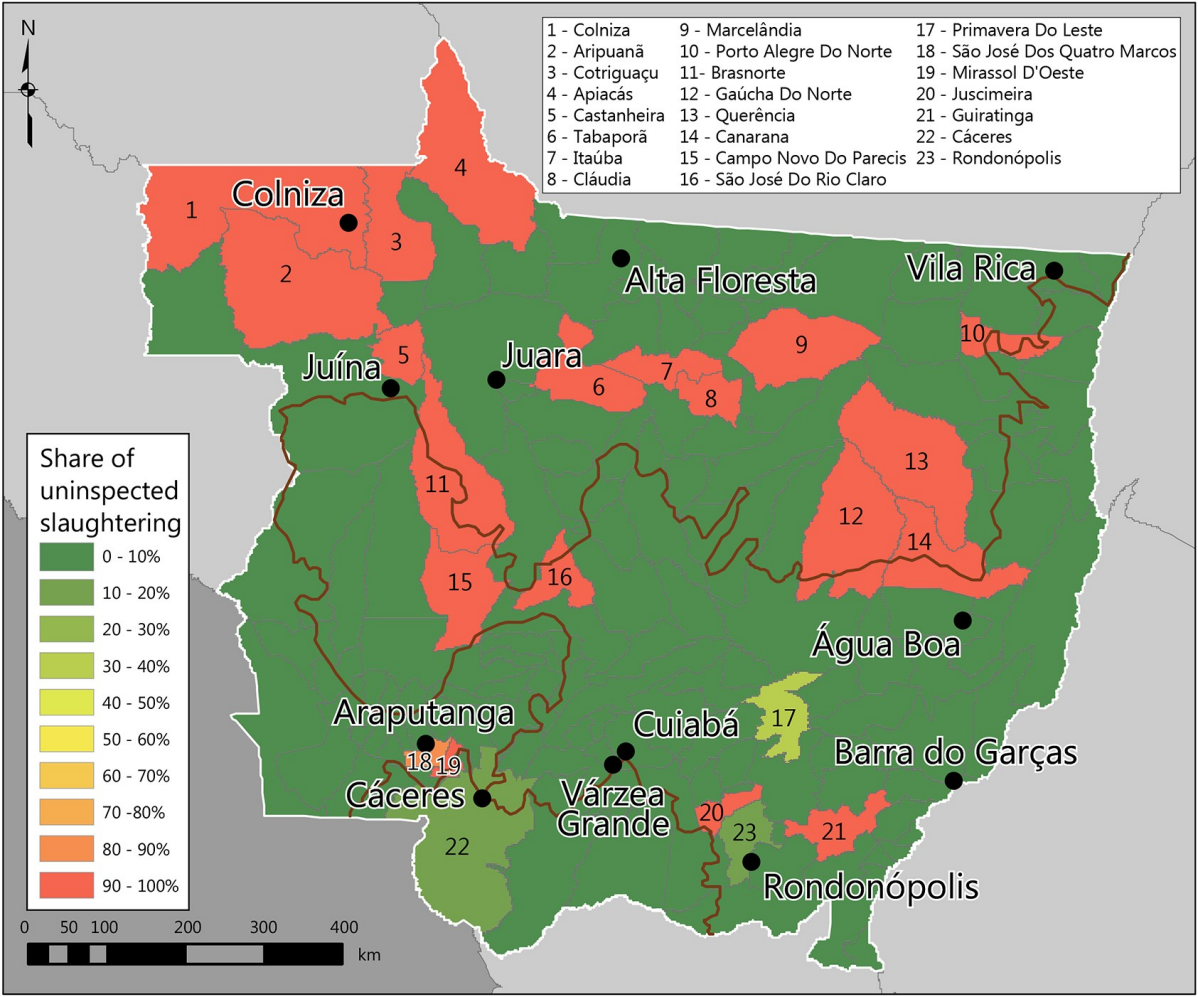
Share of uninspected slaughters to total municipality slaughters, 2016. The slaughter of animals without inspection for food safety is concentrated in a few municipalities, while most of the others have very low levels of uninspected slaughter. The locations with a high incidence of uninspected slaughter tend to have high cattle densities but a poor road connection to the main urban centers. Sources: same as in Fig 2.

The estimated volume of uninspected slaughter in Mato Grosso was 139,506 head in 2016, 3.2% of the total (Table 1). Spatially, uninspected beef products are present in municipalities across the state, with two noticeable clusters (Fig 5). The first is in the northwestern corner including both the Colniza frontier region and the more developed stretch toward Brasnorte. The second cluster is in municipalities just north of Sinop in central-northern Mato Grosso including Cláudia and Tabaporã. Both are frontier locations. However, the distribution of the uninspected slaughter among municipalities is highly polarized, with a few municipalities having almost 100% uninspected while most others have very little.

Our estimate of the uninspected share can be considered a minimum value. Even with the assumption that the few SIM plants are effectively uninspected, we are likely underestimating the size of the uninspected market. One reason is that clandestine abattoirs are absent from the slaughter records, deflating our estimate. Another reason is that even non-clandestine plants may avoid recording slaughter transactions in the inspection agency’s GTA database. This results in a further deflated estimate. When a slaughter transaction gets recorded in the GTA, the seller pays a fee and the slaughterhouse is liable to pay taxes, so both have an incentive to slaughter undocumented cattle. This incentive to slaughter cattle without a GTA is more prevalent in uninspected plants as the inspection systems (SIF and SIE) require slaughterhouses to keep a copy of the GTA documentation.

### Data validation

To start assessing the completeness of the data, we compared the slaughter volumes in the GTA records to official IBGE data, which come from a different source. While large discrepancies should suggest error, differences of a few percentage points can be attributed to the slaughter of cattle from other states, which is captured in the IBGE counts but not in the GTA. Table 1 shows that the yearly slaughter volumes in our data are between 1.4% and 3.6% below the IBGE counts, so the data appear to successfully capture slaughter volumes across the years.

Next, we evaluated the accuracy of the geolocations. In 37% of the cases, almost always where the plants are SIF or SIE, we found that the identified locations show the typical infrastructure of a slaughterhouse. In the remaining 63% of the cases, mostly for the uninspected plants, the geolocations could not be confirmed as being a slaughterhouse, either because no address was available, because the address could not be mapped to any location, or because the location did not display the features of a slaughterhouse. In these cases, we identified the town where the plant is located and took the town’s centroid as the spatial coordinate. Since the majority of plants are located at or right outside the urban areas, likely in most cases this reduces the error to a few kilometers at most.

We also measured the degree to which the grouping of CNPJs into physical plants was subject to error. During the process of compiling the present dataset a total of six new versions were produced, each of which incorporated improvements in methods as well as new data. In some cases, CNPJs previously allocated to a wrong plant were recoded to the correct plant. By computing the number of CNPJs that were recoded to a different plant between the first and the last versions of the dataset we can have an indication of the size of the error. The result is that only 3.3% of the CNPJs were recoded, half of which belong to SIF plants.

To investigate accuracy in the temporal component, we compared the time span captured by our data with historic evidence on the expansion of the meatpacking industry. Slaughter facilities were concentrated in the southern states as well as in São Paulo until the late 1970s, then started to move northward. The first periods of expansion into Mato Grosso were the late 1970s and especially the 1980s [31]. This is in line with our results. Specifically, published evidence suggests that slaughterhouses were present in Cuiabá as early as the 1970s [63]. The report of a senior veterinarian who worked for over 30 years in Mato Grosso’s sanitation inspection system confirms this. He recalled that one SIF slaughterhouse was active in 1974 in Várzea Grande, and another opened in 1975, while there were plenty of small (non-refrigerated) abattoirs at the time that were uninspected.

Our records capture the first SIF plant opening in 1967 (with a SIF registration in 1976), while the second SIF was registered in 1985, hence with a ten-year delay. [64] reported a third plant in Barra do Garças in 1974, which appears in our SIF registration records in 1978. These three SIF plants were the first refrigerated units and they processed cattle from across the state. Overall, our records estimate the starting dates of these plants with an average error of +2.3 years per plant.

Turning to the north of the state, expert consultations in the Alta Floresta region suggested that a slaughterhouse from Várzea Grande, 800 km south, slaughtered cattle from the region until the mid-1990s. Before 1996, the nearby town Colíder had one refrigerated plant, the owner of which built a plant in Alta Floresta between 1996 and 1998. Our data depict this account with relative accuracy: the Colíder plant appears in 1996 while the Alta Floresta plant appears in 1998. Moreover, the informants recalled that in the early 2000s there were five uninspected abattoirs in the Alta Floresta municipality. Our data show a pattern consistent with this account as there were six uninspected CNPJs with opening dates starting in 2005. This suggests that the abattoirs may have operated in a clandestine way for some years before becoming registered companies. Finally, the informants reported that the last uninspected abattoir stopped operating in 2017. Our GTA records show no slaughter transactions for uninspected plants after 2013, so the data do not capture the most recent dynamics of the uninspected market.

To validate the estimates for the uninspected market, we computed the number of formal workers registered in the cattle slaughter industry in municipalities with different types of plants. If uninspected slaughterhouses are small businesses, then municipalities without larger plants (SIF or SIE) should display low numbers of workers in cattle slaughter. Using data from 2016, we found that 27 municipalities with at least one SIF or SIE plant had, on average, 680 registered workers. By contrast, 96 municipalities with no slaughterhouse in our database had 0.21 registered workers in cattle slaughter, on average. Where there were only uninspected plants (18 municipalities), the average was four workers per municipality. This comparison suggests that our measure of uninspected is indeed capturing municipalities dominated by small abattoirs.

### Evolution of slaughterhouses, pasture and cattle herds over time

**Fig 6:**
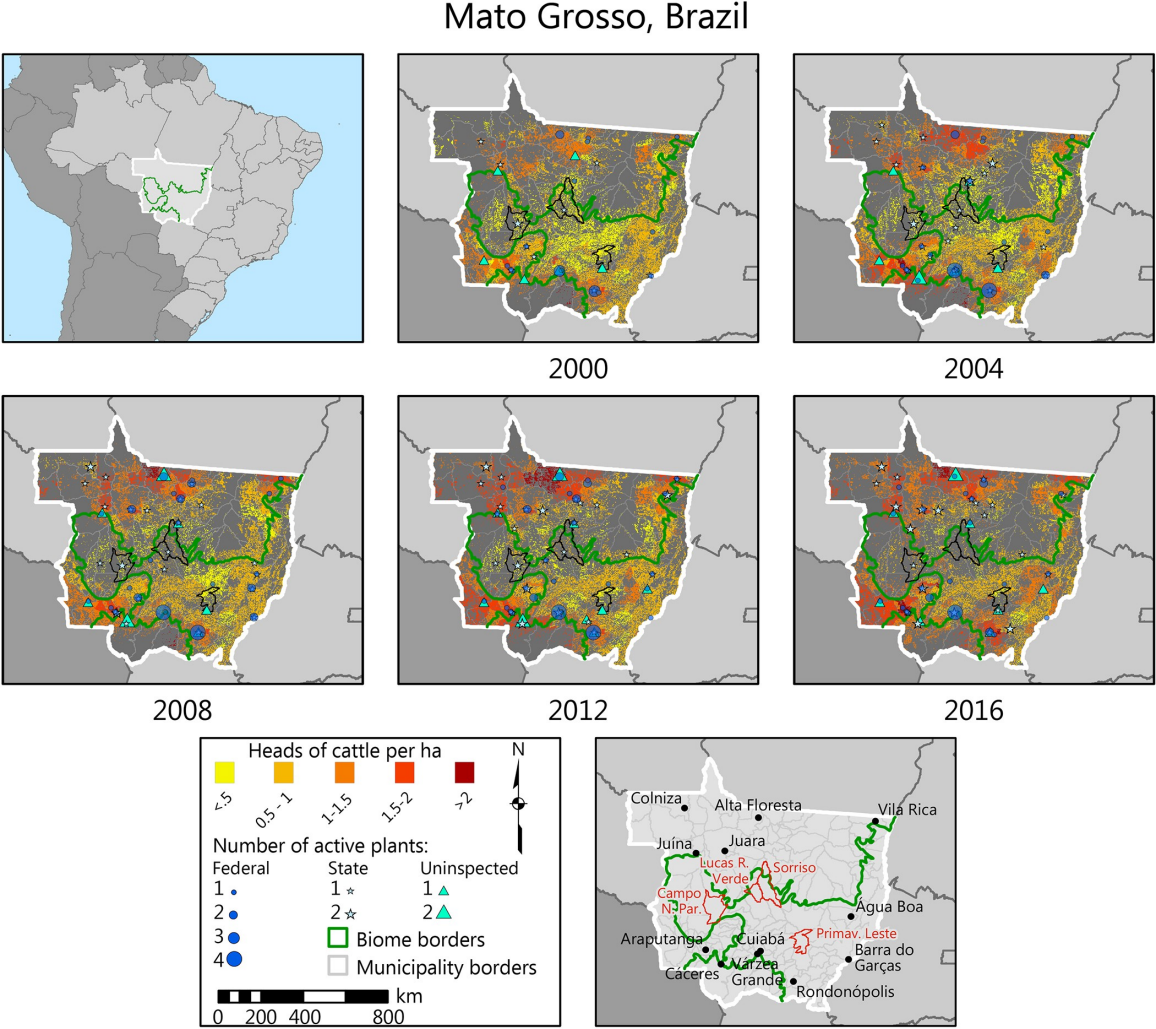
Evolution of slaughterhouses, pastures and cattle densities, 2000–2016. Cattle densities (head per hectare of pasture) calculated from [32] and the maximum pasture area in each municipality. Pastures include all pixels classified as ‘pastures’ or ‘pastures or agriculture’ by [54] or [56]. For seven plants with a closing date but without a starting date, we estimated the starting date using the average life-cycle of the plants in the same inspection category. Sources: same as in Fig 2.

In this section, we map the spread of slaughterhouses, pasturelands and cattle herds in Mato Grosso in 2000–2016. The data show almost no change in the area under pastures, which is expected given that in this period Mato Grosso saw important gains in the stocking rate of pastures and a large expansion of crop areas. Taking 3-year averages for the earliest and latest periods (2000–2002, 2014–2016), the pasture surface increased by 4%. This is in reasonable agreement with official state data summarized by [62] showing a 7.3% growth in 2002–2010 (our data yield 9.6% for this period). One reason for the slow growth in pasturelands is the rapid expansion of crop areas, although many other factors likely played a role [41, 65]. The replacement of pastures for crops was especially prevalent in the (mostly) Cerrado locations of Sorriso, Lucas do Rio Verde, Primavera do Leste, and Campo Novo do Parecis, where mechanized cropping advanced the most (Fig 6). This is confirmed by [62] for the years 2005–2010. The areas where pastures have instead expanded are the north and northeast, especially before 2012, and the Pantanal biome, after 2012.

**Fig 7:**
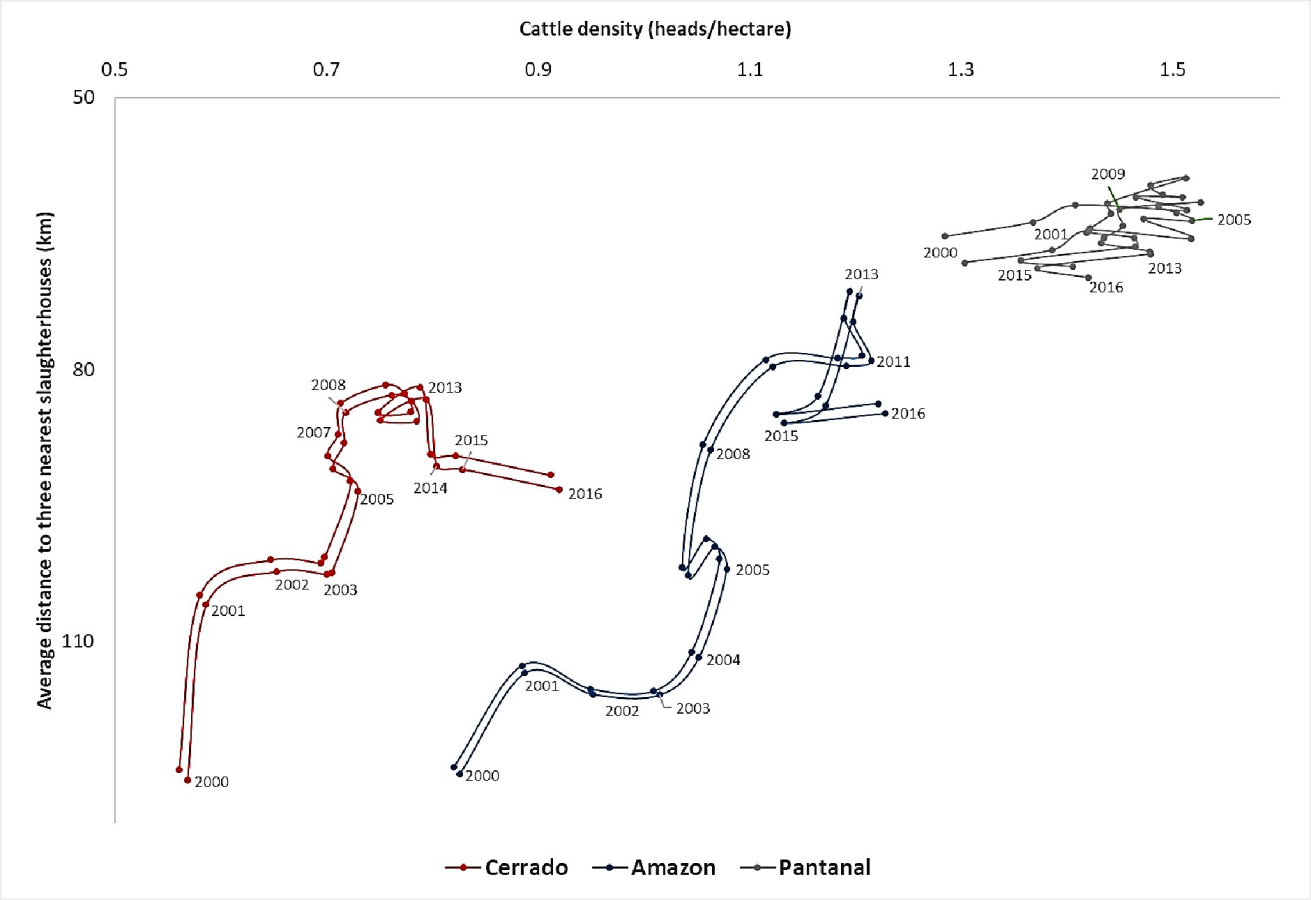
Average distance to three nearest slaughterhouses and cattle densities by biome, 2000–2016. To obtain distances between pasture areas and slaughterhouses, we generated 15,000 random points over the pasture areas and calculated the distance between each point and each slaughterhouse. The random points were replicated three times to assess robustness. Two lines are thus presented for each biome, one with the minimum and one with the maximum distance values. Cattle densities (head per hectare of pasture) were calculated from [32] and the maximum pasture area in each municipality. Pastures include all pixels classified as ‘pastures’ and ‘pastures or agriculture’ by [54] or [56]. For seven plants with a closing date but without a starting date, we estimated the starting date using the average life-cycle of the plants in the same inspection category. Sources: same as in Fig 2.

Cattle densities, on the other hand, saw a widespread increase. Using the same three-year averages, we found that head of cattle per hectare rose by 41% in 15 years. The change was more pronounced in the Cerrado biome (+62%), followed by the Amazon (+48.5%) and Pantanal (+9%) (Fig 7). The 2016 levels, however, remained lower in the Cerrado (0.91 head/ha) than in the Amazon (1.22 head/ha), where both relatively new settlements (Alta Floresta, Juína, Juara, 1970s) and old settlements (Araputanga, early 20^th^ Century) displayed high stocking rates (Fig 6). The elevated carrying capacity of pasturelands in the northern region of Alta Floresta is well known (due to a combination of high fertility, a more recent settlement, and a favorable rainfall regime) and also confirmed by [62]. The Pantanal biome, with only 4.05% of the pasture areas of the state, showed no clear temporal trend, although both the cattle stocking rate and proximity to slaughterhouses are the highest among the three biomes.

Slaughterhouses expanded especially in the north and northwest, the parts of the state where cattle densities were high and pastures were expanding, notably before 2013. A less important expansion was seen in the west, a region that remains relatively underserved.

The overall density of slaughterhouses relative to the location of pastures was mapped by computing average distances between each pasture point and the three nearest active plants. The cumulative frequencies of the average distances show a systematic decrease over the years (S2 Fig). The lowest value was reached in 2013 (76.3 km), after which the high number of plant closings negatively affected the slaughterhouse density, increasing the average distance (Fig 4). Still, between the 2000–2002 and 2014–2016 triennia, the average distance dropped by 23%. The trajectory was similar in the Cerrado and Amazon biomes (Fig 7), and there was a statistically significant negative correlation (r = -.327, p<0.001) between average distances and cattle densities.

## Discussion and conclusion

The slaughterhouse database presented in this paper provides a benchmark model to the mapping of supply chains using the triangulation of multiple digital sources, including one large database on slaughter records (GTA) that is first being used for this purpose. We developed procedures to gather, standardize, and classify information from eleven sources that amounted to several million records depicting the cattle industry in Brazil. The resulting spatial and temporal dataset is the most complete source of information on the births and deaths, locations, slaughter volumes, and company characteristics of the slaughter industry in Brazil’s Mato Grosso. It is expected that this database should open new possibilities to the study of supply chains, land use change, and the socioeconomic drivers and outcomes of the slaughter industry.

### Limitations

The use of the dataset needs to be carefully tailored against the inherent data limitations. First, the grouping of CNPJs into physical plants is error-prone, which can compromise the inferences made for the plants. However, we documented that a low rate of 3.3% of the CNPJs were grouped in the wrong plant at some point during the production of the dataset. The information derived from the GTA data are also very close to complete, as shown in the data validation, so inferences about slaughter volumes and company activity and inactivity for the years 2013–2016 are generally trustworthy.

Second, the dates of ownership change can have errors as in many cases no information was available, so inference based on reasonable assumptions had to be made. The information related to the larger holding groups is less error-prone as it was collected from sources that documented merges and acquisitions. The closing dates are the most certain after 2013, when GTA data were available. Prior to that, the further back in time the less certainty there is. In particular, no closing dates were available for years before 2002, suggesting that closings are being missed between 1967 and 2002.

The opening dates present a relatively small error rate, as shown in the data validation section. However, while our data partially capture the earliest slaughterhouse openings, it may be missing information from the 1970s through the 1990s as older company registrations are less likely to have made it to online repositories. This can compromise analyses that require historical trends for the first phase of expansion of slaughterhouses. For those cases, archival research is a more appropriate method. But for the bulk of applications involving cattle-driven land use change, the data presented here are the richest information set currently available.

Third, the locations of plants could be validated in 37% of the cases. The remaining plants were geocoded to the center of the corresponding towns. Field experience as well as the locations of the plants that could be geocoded indicate that slaughterhouses are almost always placed in the outskirts of towns. Using the centroids, therefore, likely leads to errors of a few kilometers at most. Moreover, the vast majority of the SIF locations were validated. Considering that the average distance between pasture areas and slaughterhouses is 94 km, a shift of a few kilometers in the location of SIE and uninspected plants is unlikely to affect the results substantially.

### Suggested applications

Given the limitations above, the data are best used for inferences and applications related to state-wide trends. Enquiries about specific regions, municipalities, or plants require a more in-depth approach. Research questions that can benefit from this dataset include the assessment of whether the opening and closing of slaughterhouses is a good predictor of land use change outcomes, such as the substitution of pastures for crops; or whether the location of slaughterhouses is correlated with the construction of cattle confinements, a relatively new phenomenon that is advancing toward the Amazon and changing the agricultural landscape; or about the pattern of spatial and economic agglomeration of the industry over time and its possible effects on live cattle and beef prices. Policy-relevant questions related to supply chain-monitoring can also be addressed by the data presented here, including integrating the spatial distribution of plants without zero-deforestation commitments into future versions of the agreements, assessing the correlation between the dynamics of slaughterhouses and deforestation, and using the distribution of uninspected cattle slaughtering to improve slaughterhouse sanitation standards.

### New insights

The analysis of the temporal and spatial dynamics of slaughterhouses generates new questions and insights for future studies. The finding that single plants operate through multiple active CNPJs had not, to the best of our knowledge, been previously documented in the scientific literature. What motivates slaughterhouses to operate in such a way? Three reasons can be hypothesized: 1) administrative motivations, where different CNPJs may be devoted to different subsections of the business (e.g.: domestic versus foreign markets); 2) fiscal reasons, where smaller plants split their revenues among different companies to benefit from low-taxation brackets; 3) slaughterhouses could use multiple CNPJs to explore regulatory loopholes, such as the monitoring of specific CNPJs by the authorities for fiscal or environmental enforcement.

We identified one period of slow expansion of plants until the middle 1990s, one period of rapid expansion in 1996–2003, and one period of expansion and contraction starting in 2004. The data then show a reversal of the growth trend in the number of plants starting in 2014. The high incidence of closings over the most recent years is likely to result from a combination of market concentration [48] and an economic recession. The holding group that was pivotal in the market consolidation process, JBS, was investigated for setting monopolistic prices in Mato Grosso [50], and later, hit by a sequence of corruption scandals. This created uncertainty in the industry and the expectation that a new market restructuring process could unfold.

The high market share obtained for the SIF plants in Mato Grosso is very close to what the official IBGE data depict, which serves as further validation of our estimates. Is Mato Grosso a special case in Brazil? The official data show that at least four states present a similar market share for SIFs: Mato Grosso do Sul, Rondônia, Goiás, and Tocantins [39]. The common features of these states are a large cattle supply, a low sanitation risk, and a relatively small internal demand, implying that slaughterhouses sell a large portion of their production to other markets, which only SIF plants are legally entitled to do. Consistent with this explanation, states with large human populations have a systematically lower share of SIFs, and consequently a higher share of SIEs and smaller plants: Minas Gerais, Paraná, and Rio Grande do Sul [39].

While uninspected plants are responsible for a small market share, our estimate of 2–3% is a minimum value. For example, [22] estimated a rate of 5.6% for Mato Grosso, which could imply that our figure is underestimated by 100%. This would be due to the lack of data on clandestine slaughter as well as to implicit incentives for ranchers and slaughterhouses to avoid filing slaughter transaction forms. Despite that, we show for the first time that uninspected plants are scattered across the state and that they have a shorter life-cycle than other plants (larger plants live twice as long as the smaller plants).

Finally, we mapped the expansion of slaughterhouses over the years and show that it has been significantly and positively associated with cattle densities. In a context where pasture areas grew by only 4% in 15 years and are expected to continue growing at a slow pace or even decrease in absolute size, this result makes sense. One implication is that the future dynamics of the slaughter industry, such as expansion and technological change, will be increasingly associated with intensification-prone areas such as where crop-pasture integration and cattle confinements are growing. While this paper has contributed novel data to better understand those future trajectories, more work is needed to validate the results, expand the slaughterhouse database to other states and biomes, and use the newly available digital information sources to map other relevant features of agriculture such as confinements and cattle ranching intensification.

## Supporting information

S1 Supporting information A. Portuguese abstract. B. Data access.
(DOCX)

S1 Table Data attributes and sources. (DOCX)

S2 Table Counts and age of plants and CNPJs, 2016. **-** Sources: [58]; company registry (CNPJ), Empresômetro; Sintegra; Taxpayer Central Registry; Ministry of Agriculture (see S1 Table for more details on sources).
(DOCX)

S1 Box Cattle slaughter records, Indea-MT. (DOCX)

S1 Fig Maximum pasture area and 15,000 random points, 2000–2016. Pastures include all pixels classified as ‘pastures’ or ‘pastures or agriculture’ by [54] or [55] at any year between 2000 and 2016.
(DOCX)

S2 Fig Cumulative frequencies of average distance to three nearest slaughterhouses, 2000–2016. Cattle densities (head per hectare of pasture) are calculated from [32] and the maximum pasture area in each municipality. Pastures include all pixels classified as ‘pastures’ and ‘pastures or agriculture’ by [54] or [55]. For seven plants with a closing date but without a starting date, we estimated the starting date using the average life-cycle of the plants in the same inspection category.
Sources: [23,24,32,54,55,58]; company registry (CNPJ), Empresômetro; Sintegra; Taxpayer Central Registry; Ministry of Agriculture; (see S1 Table for more details on sources).
(DOCX)
