# Supplementary material for: Mapping the cattle industry in Brazil’s most dynamic cattle-ranching state: Slaughterhouses in Mato Grosso, 1967-2016
Source: PLoS One. 2019 Apr 30;14(4):e0215286. doi: 10.1371/journal.pone.0215286 (PMC6490905; doi:10.1371/journal.pone.0215286)
Supplement: S1 Supporting information — A. Portuguese abstract. B. Data access. (DOCX) [file pone.0215286.s001.docx]

**Mapping the cattle industry in Brazil’s most dynamic cattle-ranching state: slaughterhouses in Mato Grosso, 1967-2016**

Petterson Vale^12^*, Holly Gibbs^1^, Ricardo Vale^12^, Jacob Munger^1^, Amintas Brandão Jr.^1^, Matthew Christie^1^, Eduardo Florence^13^

*Corresponding author ([p.m.vale@alumni.usp.br](mailto:pvale@wisc.edu)) (PV)

^1^Nelson Institute for Environmental Studies, Center for Sustainability and the Global Environment (SAGE), University of Wisconsin-Madison, Madison (WI), USA

^2^Department of Economics, University of São Paulo, Ribeirão Preto (SP), Brazil

^3^Laboratory of Soils, State University of Mato Grosso, Alta Floresta (MT), Brazil

**Supporting Information**

**A. Portuguese Abstract**

**Resumo**

O Mato Grosso é o maior produtor bovino do Brasil com um rebanho de 30,2 milhões de cabeças em 2017. Com um padrão de uso do solo fortemente influenciado pela pecuária, atividade que ocorre em largas porções de terra, o estado é um dos principais alvos para a conservação ambiental no Brasil. No entanto, a dinâmica espacial e temporal da indústria do abate em Mato Grosso é desconhecida devido a limitações dos dados. Neste estudo, criamos um novo método para mapear a expansão e a contração do setor do abate bovino. Analisamos a abertura e o fechamento de 133 frigoríficos entre 1967 e 2016 em Mato Grosso, estimando as suas localizações e volumes de abate. Isso foi feito por meio da triangulação de diferentes bases de dados, entre as quais uma lista de 21 milhões de empresas, registros de 3 milhões de transações de abate (a partir de GTAs – Guias de Trânsito Animais), e imagens de satélite de alta resolução. Este estudo é o primeiro a incluir informação longitudinal e plantas inspectionadas e não inspecionadas (para sanidade alimentar). Os resultados mostram que 72 plantas operavam em 2016 por meio de 52 empresas. A densidade de plantas ativas relativamente às áreas de pastagem cresceu 29% em 2000-2016, demonstrando uma expansão da infraestrutura de abate. Identificamos três períodos de expansão: 1967-1995, com 15,1% das aberturas de plantas; 1996-2003, com 24,6%; e 2004-2016, com 60,3%. Ao passo que os fechamentos ocorreram provavelmente durante todo o período estudado, não houve dados para fechamentos anteriores a 2002. Estimamos em 2-3% o volume de abate não-inspecionado para sanidade alimentar em 2013-2016. Concluímos o artigo discutindo potenciais aplicações dos dados, que estão disponibilizados sem identificação de empresas em um repositório online. O método aqui desenvolvido pode ser replicado para todo o território nacional, ampliando o nosso entendimento sobre a dinâmica do abate bovino e seu impacto sobre o uso do solo.

**Data access.** A deidentified version of the data generated in this paper is freely available at <http://doi.org/10.3886/E106982V1> [57]. Please contact the authors to discuss access to a more complete version of the data.

**References**

23. Barreto P, Pereira R, Brandão JA, Baima S. Os frigoríficos vão ajudar a zerar o desmatamento da Amazônia? [internet] Belém: Imazon; 2017 [cited 2018 May 15]. Available from: http://www.imazon.org.br/PDFimazon/Portugues/livros/Frigorificos%20e%20o%20desmatamento%20da%20Amaz%C3%B4nia.pdf.

24. LAPIG – Laboratório de Processamento de Imagens e Geoprocessamento. Matadouros e frigoríficos do Brasil, 2014. Goiânia (Brazil): Lapig-Maps; 2016 [cited 2016 Jun 24]. Available from: http://maps.lapig.iesa.ufg.br/lapig.html

32. IBGE – Instituto Brasileiro de Geografia e Estatística [dataset]. Pesquisa Pecuária Municipal; 2017 [cited 2017 Oct 22]. Avaliable from: https://sidra.ibge.gov.br/pesquisa/ppm/quadros/brasil/2016https://sidra.ibge.gov.br/pesquisa/ppm/quadros/brasil/2016

48. Carvalho, TB. Estratégias de crescimento e reestruturação da indústria de carne bovina no Brasil: o papel de políticas públicas discricionárias [dissertation]. São Paulo (Brazil): Department of Business Administration, University of São Paulo; 2016.

50. DOE-ALE/MT – Diário Oficial do Estado, Assembleia Legislativa de Mato Grosso. Relatório final da comissão parlamentar de inquérito dos frigoríficos [internet]. Cuiabá (Brazil): Assembleia Legislativa de Mato Grosso; 2016 [cited 2017 Sep 9]. Available from: <https://www.al.mt.gov.br/storage/webdisco/cp/20161214093952144400.pdf>

54. MapBiomas [internet]. Project MapBiomas – Collection 2.3 of Brazilian Land Cover & Use Map Series; 2017 [cited 2018 Feb 10]. Available from: http://mapbiomas.org/

55. Parente L, Ferreira L, Faria A, Nogueira S, Araújo F, Teixeira L, Hagen S. Monitoring the razilian pasturelands: A new mapping approach based on the Landsat 8 spectral and temporal domains. International Journal of Applied Earth Observation and Geoinformation 2017;62:135-43.

58. Indea – Instituto de Defesa Agrossilvopastoril de Mato Grosso [internet]. Cuiabá (Brazil): Consulta de GTA; 2017 [cited 2018 Apr 4]. Available from: <http://sistema.indea.mt.gov.br:8082/SIA/form.jsp?sys=SCA&action=openform&formID=467&align=0&mode=-1&goto=-1&filter=&scrolling=no>
