# Supplementary material for: Mapping the cattle industry in Brazil’s most dynamic cattle-ranching state: Slaughterhouses in Mato Grosso, 1967-2016
Source: PLoS One. 2019 Apr 30;14(4):e0215286. doi: 10.1371/journal.pone.0215286 (PMC6490905; doi:10.1371/journal.pone.0215286)
Supplement: S1 Table — (DOCX) [file pone.0215286.s002.docx]

**S1 Table. Data attributes and sources**

| **Field** | **Details** | **Data source** | **Web link** |
| --- | --- | --- | --- |
| Municipality | _ | CNPJ registry compiled by Empresômetro | [www.empresometro.com.br](http://www.empresometro.com.br) |
| Holding name | Most recent proprietor | CNPJ registry; [48,50] | [www.empresometro.com.br](http://www.empresometro.com.br) |
| Plant name | Commercial name for physical plant | CNPJ registry, Empresômetro | [www.empresometro.com.br](http://www.empresometro.com.br) |
| CNPJ number | 14-digit company identifier | CNPJ registry, Empresômetro | [www.empresometro.com.br](http://www.empresometro.com.br) |
| Opening date | Federal inspection system or CNPJ was first registered | Ministry of Agriculture; CNPJ registry; Sintegra; Indea-MT (GTA) [58] | [www.agricultura.gov.br/assuntos/inspecao/produtos-animal/sif](http://www.agricultura.gov.br/assuntos/inspecao/produtos-animal/sif) |
| Update date | CNPJ information was updated | Sintegra | www.sintegra.gov.br |
| Closing date | Company was discontinued at state-level | Sintegra; Indea-MT (GTA) [58] | www.sintegra.gov.br  www.indea.mt.gov.br |
| Holding date | Ownership change: CNPJ was absorbed by the current holding group | DOE-ALE/MT (2016); Carvalho (2016) [48,50]; authors’ inference | _ |
| Active CNPJ | The company registration has an ‘active’ status | Sintegra; Taxpayer Central Registry (CCC) | www.sintegra.gov.br  www.sefaz.rs.gov.br/NFE/NFE-CCC.aspx |
| Plant type | Federal or state inspection | Ministry of Agriculture (MAPA); Indea-MT (GTA) [58] | [www.agricultura.gov.br/assuntos/inspecao/produtos-animal/sif](http://www.agricultura.gov.br/assuntos/inspecao/produtos-animal/sif) |
| G4 status | Zero-deforestation voluntary commitment | G4 cattle agreement | _ |
| TAC status | Zero-deforestation agreement with Federal Prosecutors | Federal Prosecutors' Office (MPF) | www.mpf.mp.br/mt/sala-de-imprensa/pecuaria-sustentavel |
| Yearly slaughter | Total head slaughtered, 2013-2016 | Indea-MT (GTA) [58] | [www.indea.mt.gov.br](http://www.indea.mt.gov.br) |
| Name variations | Legal names that appear attached to a single CNPJ | CNPJ registry compiled by Empresômetro; Indea-MT (GTA) [58] | [www.empresometro.com.br](http://www.empresometro.com.br)  [www.indea.mt.gov.br](http://www.indea.mt.gov.br) |
| Address | _ | CNPJ registry, Empresômetro | [www.empresometro.com.br](http://www.empresometro.com.br) |
| Spatial coordinates | Latitude, Longitude | GPS coordinates collected in-situ; Google enterprise registry; Barreto et al. (2017) and LAPIG (2016) [23,24]; visual inspection of CNPJ registry’s address; centroid of municipality | [www.agricultura.gov.br/assuntos/inspecao/produtos-animal/sif](http://www.agricultura.gov.br/assuntos/inspecao/produtos-animal/sif)  [www.abiec.com.br/MapaDasPlantas.aspx](http://www.abiec.com.br/MapaDasPlantas.aspx)  www.google.com/maps/ |
