# Supplementary material for: Mapping the cattle industry in Brazil’s most dynamic cattle-ranching state: Slaughterhouses in Mato Grosso, 1967-2016
Source: PLoS One. 2019 Apr 30;14(4):e0215286. doi: 10.1371/journal.pone.0215286 (PMC6490905; doi:10.1371/journal.pone.0215286)
Supplement: S2 Table — - Sources: [58]; company registry (CNPJ), Empresômetro; Sintegra; Taxpayer Central Registry; Ministry of Agriculture (see S1 Table for more details on sources). (DOCX) [file pone.0215286.s003.docx]

**S2 Table. Counts and age of plants and CNPJs, 2016**

|  | Active plants^1^ | | Inactive plants^2^ | | CNPJs | |
| --- | --- | --- | --- | --- | --- | --- |
| Plant type | Count | Average age (years) | Count | Average age (years) | Per plant | Average age (years) |
| Federal (SIF) | 30 | 18.4 | 12 | 9.5 | 2.3 | 9.1 |
| State (SIE) | 9 | 13.6 | 4 | 7.8 | 2.6 | 10.0 |
| Uninspected^3^ | 33 | 8.1 | 45^a^ | 5.6 | 1.1^b^ | 7.6 |
| Total | 72 | 13.2 | 61 | 6.9 | 2.0 | 8.7 |

^1^Plants with positive slaughter activity in 2016. ^2^Plants with no slaughter activity in 2016. ^3^All slaughterhouses without a federal inspection or state inspection code were defined as uninspected. ^a^Includes seven inactive plants with a missing average age. ^b^Excludes plants coded as ‘unidentified’, where no name or address could be found in the CNPJ records.

Sources: [58]; company registry (CNPJ), Empresômetro; Sintegra; Taxpayer Central Registry; Ministry of Agriculture (see S1 Table for more details on sources).
