# Supplementary material for: Mapping the cattle industry in Brazil’s most dynamic cattle-ranching state: Slaughterhouses in Mato Grosso, 1967-2016
Source: PLoS One. 2019 Apr 30;14(4):e0215286. doi: 10.1371/journal.pone.0215286 (PMC6490905; doi:10.1371/journal.pone.0215286)
Supplement: S1 Box — (DOCX) [file pone.0215286.s004.docx]

**S1 Box. Cattle slaughter records, Indea-MT**

Animal Transit Forms (GTAs) are documents that must be filed for every transportation event of livestock animals (bovine cattle, poultry, fish, and others) in Brazil. These traceability data are collected exclusively to support public health by preventing the spread of foot-and-mouth and other animal diseases. A form with a unique seven-digit code is generated for each load of animals that is transported; this could be a truck load, a boat load, or a herd being moved by foot. Fixed or moving fiscal agents can request to see the form, and there are penalties for animals being transported unregistered. The GTA forms are recorded in databases managed by the state sanitation agencies in Brazil and synchronized with a federal database. The following information are included in the data: quantity, sex, number of animals within different age ranges, purpose of the transportation (slaughter, breeding, etc), origin and destination municipalities and properties, and vaccination status of the animals.

The GTA data are a key component of Brazil’s animal disease traceability program, which is regulated by Federal Law 12.097/2009 and Federal Decree 7.623/2011. Access to the data from federal servers is further regulated by the Ministry of Agriculture’s normative instruction 23/2015. All data from government sources in Brazil are also regulated by the Federal Information Access Law 12.527/2011, which allows the use of personal information in special cases, including scientific research and statistical analysis subject to personal information not being made public.

GTA forms for the years 2013 to 2017 were downloaded from public servers in Brazil [58] by the Gibbs Land Use and the Environment Laboratory at the University of Wisconsin-Madison. The data used in this paper are from the gibbs database version 3.4.4.
