# Supplementary material for: Mapping the cattle industry in Brazil’s most dynamic cattle-ranching state: Slaughterhouses in Mato Grosso, 1967-2016
Source: PLoS One. 2019 Apr 30;14(4):e0215286. doi: 10.1371/journal.pone.0215286 (PMC6490905; doi:10.1371/journal.pone.0215286)
Supplement: S1 Fig — Pastures include all pixels classified as ‘pastures’ or ‘pastures or agriculture’ by [54] or [55] at any year between 2000 and 2016. (DOCX) [file pone.0215286.s005.docx]

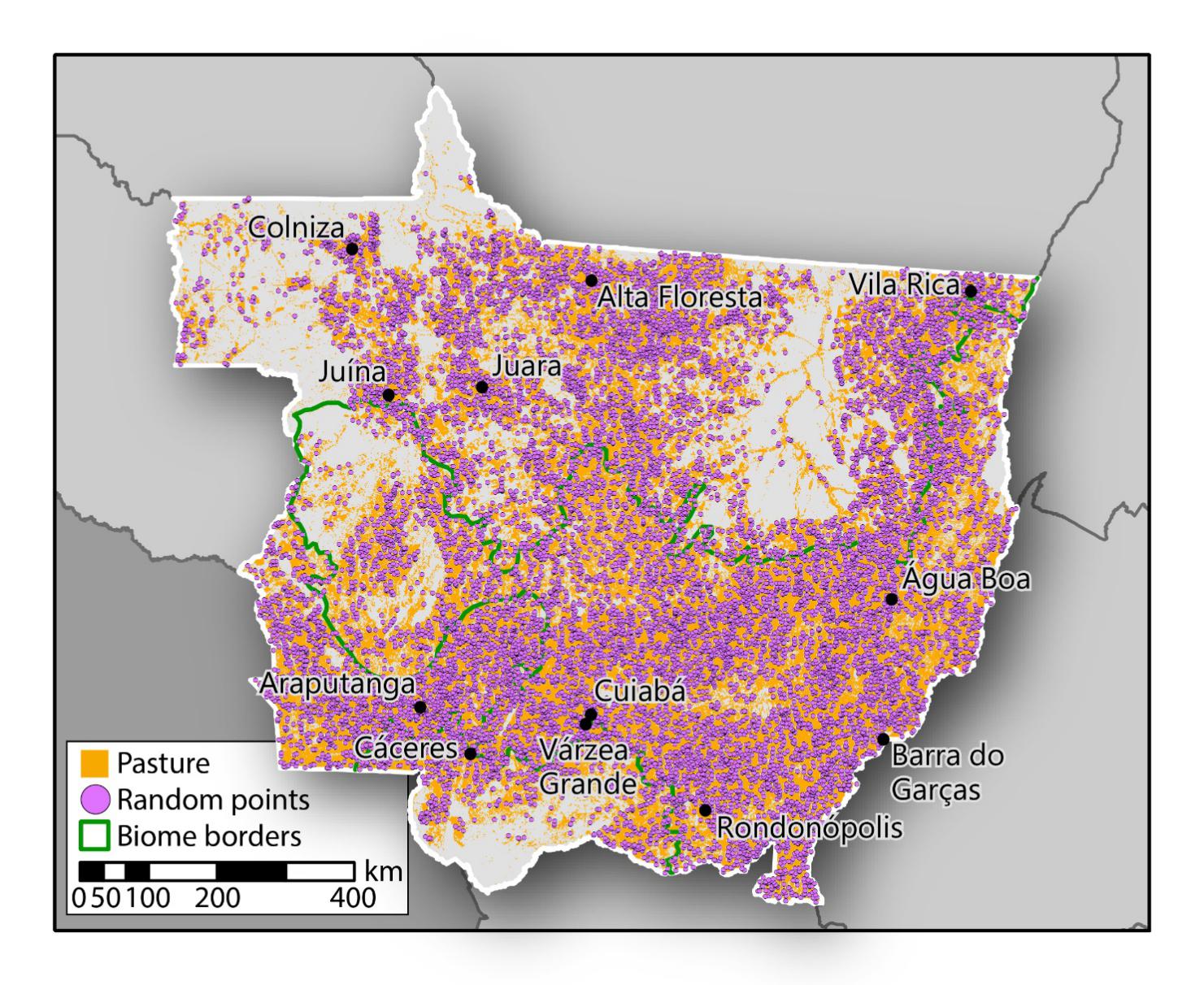


**S1 Fig. Maximum pasture area and 15,000 random points, 2000-2016.** Pastures include all pixels classified as ‘pastures’ or ‘pastures or agriculture’ by [54] or [56] at any year between 2000 and 2016.
