# Supplementary material for: Mapping the cattle industry in Brazil’s most dynamic cattle-ranching state: Slaughterhouses in Mato Grosso, 1967-2016
Source: PLoS One. 2019 Apr 30;14(4):e0215286. doi: 10.1371/journal.pone.0215286 (PMC6490905; doi:10.1371/journal.pone.0215286)
Supplement: S2 Fig — Cattle densities (head per hectare of pasture) are calculated from [32] and the maximum pasture area in each municipality. Pastures include all pixels classified as ‘pastures’ and ‘pastures or agriculture’ by [54] or [55]. For seven plants with a closing date but without a starting date, we estimated the starting date using the average life-cycle of the plants in the same inspection category. Sources: [23,24,32,54,55,58]; company registry (CNPJ), Empresômetro; Sintegra; Taxpayer Central Registry; Ministry of Agriculture; (see S1 Table for more details on sources). (DOCX) [file pone.0215286.s006.docx]

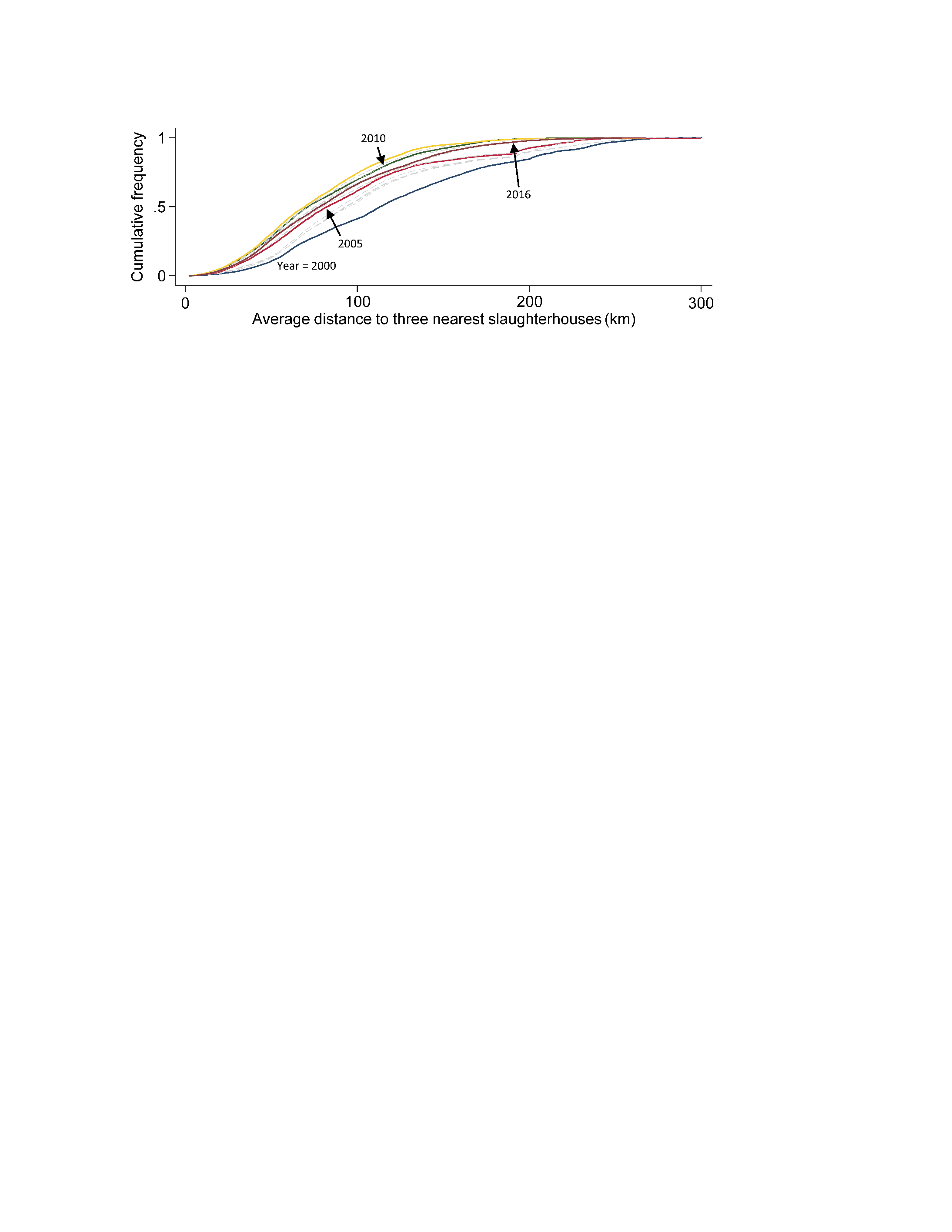


**S2 Fig. Cumulative frequencies of average distance to three nearest slaughterhouses, 2000-2016.** Cattle densities (head per hectare of pasture) were calculated from [32] and the maximum pasture area in each municipality. Pastures include all pixels classified as pastures and ‘pastures or agriculture’ by [54] or [56]. For seven plants with a closing date but without a starting date, we estimated the starting date using the average life-cycle of the plants in the same inspection category.

Sources: [23,24,32,54,56,58]; company registry (CNPJ), Empresômetro; Sintegra; Taxpayer Central Registry; Ministry of Agriculture; (see S1 Table for more details on sources).
